# Simple protocol for population (Sanger) sequencing for Zika virus genomic regions

**DOI:** 10.1590/0074-02760170248

**Published:** 2017-11-27

**Authors:** Gabriela Bastos Cabral, João Leandro de Paula Ferreira, Renato Pereira de Souza, Mariana Sequetin Cunha, Adriana Luchs, Cristina Adelaide Figueiredo, Luís Fernando de Macedo Brígido

**Affiliations:** 1 Instituto Adolfo Lutz, Centro de Virologia, Núcleo de Doenças Sanguíneas e Sexuais, Laboratório de Retrovirus, São Paulo, SP, Brasil; 2 Instituto Adolfo Lutz, Centro de Virologia, Núcleo de Transmissão Vetorial, São Paulo, SP, Brasil; 3 Instituto Adolfo Lutz, Centro de Virologia, Núcleo de Doenças Entéricas, São Paulo, SP, Brasil; 4 Instituto Adolfo Lutz, Centro de Virologia, Núcleo de Doenças Respiratórias, São Paulo, SP, Brasil

**Keywords:** Zika virus, Sanger sequencing, PCR, phylogeny

## Abstract

**BACKGROUND:**

A number of Zika virus (ZIKV) sequences were obtained using Next-generation sequencing (NGS), a methodology widely applied in genetic diversity studies and virome discovery. However Sanger method is still a robust, affordable, rapid and specific tool to obtain valuable sequences.

**OBJECTIVE:**

The aim of this study was to develop a simple and robust Sanger sequencing protocol targeting ZIKV relevant genetic regions, as envelope protein and nonstructural protein 5 (NS5). In addition, phylogenetic analysis of the ZIKV strains obtained using the present protocol and their comparison with previously published NGS sequences were also carried out.

**METHODS:**

Six Vero cells isolates from serum and one urine sample were available to develop the procedure. Primer sets were designed in order to conduct a nested RT-PCR and a Sanger sequencing protocols. Bayesian analysis was used to infer phylogenetic relationships.

**FINDINGS:**

Seven complete ZIKV envelope protein (1,571 kb) and six partial NS5 (0,798 Kb) were obtained using the protocol, with no amplification of NS5 gene from urine sample. Two NS5 sequences presented ambiguities at positions 495 and 196. Nucleotide analysis of a Sanger sequence and consensus sequence of previously NGS study revealed 100% identity. ZIKV strains described here clustered within the Asian lineage.

**MAIN CONCLUSIONS:**

The present study provided a simple and low-cost Sanger protocol to sequence relevant genes of the ZIKV genome. The identity of Sanger generated sequences with published consensus NGS support the use of Sanger method for ZIKV population studies. The regions evaluated were able to provide robust phylogenetic signals and may be used to conduct molecular epidemiological studies and monitor viral evolution.

Zika virus (ZIKV) is a member of the genus Flavivirus, of the Flaviviridae family. ZIKV was first isolated in Uganda in 1947 from a sentinel rhesus monkey. Since then, only sporadic cases of human infection and isolation from mosquitoes of the genus *Aedes* has been reported in Africa and Asia. ZIKV has been considered an emergent pathogen since 2007, when an epidemic was reported in Micronesia ( Faria et al. 2016 ).

ZIKV was first identified in the Americas in March 2015 during an outbreak of an exanthematic disease in the state of Bahia, Brazil ( Campos et al. 2015 ). In September 2015, an increase in the number of infants born with microcephaly was observed in areas where ZIKV had been previously reported, and by mid-February 2016, more than 4300 cases of microcephaly had been notified in the country. Due to technical limitations in serological tests, biological confirmation of ZIKV infection is based mostly on detection of viral RNA in serum/plasma or urine by real-time-polymerase chain reaction (qRT-PCR). It is well known that ZIKV RNA is detectable in urine at a higher load and with a longer duration than in serum ( Gourinat et al. 2015 ). Specific antibody detection is mostly hampered due to serological cross-reactivity with other circulating flaviviruses such as dengue virus or yellow fever virus ( Lanciotti et al. 2008 , Tappe et al. 2014 , 2015 ).

Recently studies on molecular epidemiology supports the hypothesis that the Brazilian ZIKV strains belong to the Asian lineage ( Faria et al. 2016 ). Genetic and genomic evaluation is important to viral evolution knowledge, vaccine development, improvement of diagnostic assays, as well as contribute to the understating on non-vectorial transmissions pathways, including sexual transmission ( Barjas-Castro et al. 2016 , Bonaldo et al. 2016 ). Next-generation sequencing (NGS) is commonly used in quasiespecies diversity studies, especially of RNA viruses, and a powerful tool for phylogenetic studies, generating consensus of major variant sequences. ZIKV sequences have been obtained using this methodology ( Behura et al. 2016 , Gu et al. 2017 ). Nevertheless, major variant sequences can also be obtained using conventional Sanger sequencing. The aim of this study was to develop a simple and robust Sanger sequencing protocol targeting relevant genetic regions of the ZIKV, as envelope protein and nonstructural protein 5 (NS5). In addition, phylogenetic analysis of the ZIKV strains obtained using the present protocol and their comparison with previously published NGS sequences were also carried out.

## MATERIALS AND METHODS

*Samples* - Six cell culture supernatants obtained from serum samples (see below) and one urine sample were available to develop the protocol. All samples were previously tested by qRT-PCR ( Lanciotti et al. 2008 ) that confirmed ZIKV infection. Two of the six samples were obtained from a donor (isolate BR17829) and recipient (isolate BR22482) pair of a reported ZIKV transmission through blood transfusion ( Barjas-Castro et al. 2016 ). One serum sample and the urine sample pair were from a same patient (isolate BR31016), with three serum samples from other, unrelated patients (isolates BR18147/ZH100, BR19147/23101702 and BR2716). The serum samples (20 μL) were first inoculated in C6/36 cell lineage ( *Aedes albopictus* cells, ATCC-CRL-1660) in order to replicate flaviviruses to high titers ( Ciota et al. 2007 ). Cell cultures were incubated for nine days at 28ºC. Indirect immunofluorescent antibody (IFA) tests were performed using flavivirus polyclonal antibodies as described by Gubler et al. (1984) in order to confirm ZIKV infection. These isolates were storage in -70ºC. The isolates obtained from C6/36 cells were then inoculated in Vero cells (African green monkey kidney cells, ATCC-CRL-81) and incubated at 37ºC with CO_2_ 5%. The tubes were observed daily, and when a cytopathogenic effect was observed, the supernatants were used to conduct the molecular assays.

*Primers design* - In order to design primer suitable PCR sets to entire ZIKV envelope protein and partial NS5, 15 sequences (KU647676, KU509998, KU681082, KJ776791, KR815990, KR815989, KR816336, KU497555, KU365778, KU365777, KU365779, KU365780, KU232301, KU232300 and KU232298) of ZIKV were obtained from NCBI and imported into BioEdit sequence alignment editor (version 7.0.5.2) program. The process of primer designing was conducted manually, and no automated software packages were used.

The primers were design to conduct a nested reverse transcription-polymerase chain reaction (RT-PCR) protocol. For ZIKV envelope protein amplification the primers set used were: (i) First round (one-step RT-PCR) Zika1_out_Forward AGCAGCAGCTGCCATCGCTTG (777-797bp) and Zika2_out_Reverse GTACCT GTCCCTCCAGGCTTC (2478-2458pb), resulting in a 1,701 kb product; and (ii) Second round (nested PCR) Zika3_Inner_Foward GATACTGCTGATTGCCCCGGCATA (843-866pb) and Zika4_Inner_Reverse TTCTTTGAGAAGTCCACCGAGCAC (2414-2391pb), generating a fragment of 1,571 Kb. These primers pair allowed the amplification of the entire ZIKV envelope protein, comprising nucleotide position 873-2370 based on MR-766 strain (accession number NC_012532) ( Kuno & Chang 2007 ).

ZIVK NS5 protein amplification used an (i) outer primer pair (one step RT-PCR) Zika1_out_foward TGAGAGGAGAGTGCCAGAGT (8891-8910pb) and Zika2_out_reverse ATAAAGGAGCTGCCACATTTG (9843-9864pb), producing a 0,973 kb fragment; and (ii) inner pair (nested PCR) Zika3_inner_foward TGGAAAGGCCAAGGGCAGC (8958-8976pb) and Zika4_inner_Reverse GTGGCGGCAGGGAACCACAAT (9736-9756pb), generating a fragment of 0,798 Kb. These pair of primers permitted the partial amplification of the NS5 protein, comprising nucleotide position 8958-9756 based on MR-766 strain (accession number NC_012532) ( Kuno & Chang 2007 ).

*Nucleic acid extraction* - ZIKV RNA was extracted from both Vero cell culture and urine by (QIAmp® viral RNA mini kit (Qiagen, Hilden, Germany) according manufacture’s protocol. Urine was extracted in duplicate: directly from sample and after concentration of 1 mL of urine by centrifugation (21,000 x g) for 1 h at 4ºC.

*RT-PCR and nested PCR protocols* - In ZIKV envelope protein one-step RT-PCR, extracted RNA was reverse-transcribed and amplified using SuperScript® III One-step RT-PCR system with Platinum Taq High Fidelity (Life Technologies, USA). The total reaction mixture volume of 50 μL contained the following: 2x reaction mix (25 μL), 10 μM primers (1 μL each), enzyme mix (reverse transcriptase and Taq polymerase, 1 μL), extracted viral RNA template (10 μL), and RNase-free water (12 μL). RT-PCR conditions for envelope amplification were as follows: reverse transcription at 55ºC for 30 min, initial PCR activation at 94ºC for 5 min, 18 amplification cycles of denaturation at 94ºC for 30 s, annealing at 56ºC for 30 s, extension at 68ºC for 2 min 30 s, 17 amplifications cycles of denaturation at 94ºC for 30 s, annealing at 60ºC for 30 s, extension at 68ºC for 2 min 30 s (a total of 35 amplification cycles), and a final extension at 68ºC for 10 min. For nested PCR, the RT-PCR product (2,5 µL), 10 μM primers (1 µL each), and RNase-free water (8 µL) were added to a Go Taq® Green Master Mix 2X (12,5 µL) (Promega Biosciences, CA). PCR conditions were as follows: initial denaturation at 94ºC for 3 min, 35 cycles of denaturation at 94ºC for 30 s, annealing at 55ºC for 30 s extension at 72ºC for 2 min, and a final extension at 72ºC for 10 min.

In ZIKV NS5 genomic region one-step RT-PCR, extracted RNA was reverse-transcribed and amplified using SuperScript® III One-step RT-PCR system with Platinum Taq High Fidelity (Life Technologies, USA). The total reaction mixture volume of 50 μL contained the following: 2x reaction mix (25 μL), 10 μM primers (1 μL each), enzyme mix (reverse transcriptase and Taq polymerase, 1 μL), extracted viral RNA template (10 μL ), and RNase-free water (12 μL). RT-PCR conditions for NS5 amplification were as follows: reverse transcription at 55ºC for 30 min, initial PCR activation at 94ºC for 5 min, 35 amplification cycles of denaturation at 94ºC for 30 s, annealing at 53ºC for 30 s, extension at 68ºC for 1 min 30 s, and a final extension at 68ºC for 10 min. For nested PCR, the RT-PCR product (2,5 µL), 10 μM primers (1 µL each), and RNase-free water (8 µL) were added to a Go Taq® Green Master Mix 2X (12,5 µL) (Promega Biosciences, CA). PCR conditions were as follows: initial denaturation at 94ºC for 3 min, 35 cycles of denaturation at 94ºC for 30 s, annealing at 58ºC for 30 s extension at 72ºC for 2 min, and a final extension at 72ºC for 10min.

The products of RT-PCR and nested PCR were loaded onto a 1.5% agarose gel and visualised under ultraviolet light.

*Sequencing* - The 1,571 Kb PCR product (complete protein) of ZIKV envelope protein amplification was sequenced using eight primers. Four primers were designed to sequencing the ~800 bp fragment obtained from partial NS5 region amplification ( Table ). Each sequencing reaction was performed using 0,5 μL of BigDye Terminator v3.1 cycle sequencing kit (Applied Biosystems) and 1,6 μL for each primer (1 µM) in 10 μL final volume per reaction. Dye-labelled products were sequenced using an ABI 3130 sequencer (Applied Biosystems). Sequencing chromatograms were edited manually using Sequencher 4.7 software (Gene Codes, USA).

**TABLE:** Zika virus (ZIKV) envelope and NS5 primer sets designed for Sanger sequencing

Primers	Sequence (5´- 3´)	Location	Region	Polarity
EZ3IFS	gAT ACT gCT gAT TgC CCC ggC ATA	843 - 866	Envelope	+
EZ5IFS	ATg ACC ggg AAg AgC ATC CAg	1243 - 1263	Envelope	+
EZ6IFS	Agg CAA ACT gTC gTg gTT CTA	1624 - 1644	Envelope	+
EZ7IFS	CTT ACA TTg TCA TAg gAg TCg	2024 - 2044	Envelope	+
EZ4IRS	TTC TTT gAg AAg TCC ACC gAg CAC	2414 – 2391	Envelope	-
EZ8IRS	g TCC CCA AAT ggT ggA TCA AgT	2001 - 2022	Envelope	-
EZ9IRS	TgC gTC CTT gAA CTC TAC CAg	1594 - 1614	Envelope	-
EZ10IRS	CT CCC TTT gCC AAA AAg TCC ACA	1183 - 1205	Envelope	-
ZNS3IFS	Tgg AAA ggC CAA ggg Cag C	8958 - 8976	NS5	+
ZN5IFS	CAg TCA gTg gAg ATg ATT gC	9542 - 9562	NS5	+
ZNS4IRS	gTg gCg gCA ggg AAC CAC AAT	9736 - 9756	NS5	-
ZN6IRS	TgT CCg CTC CCC CTT Tgg TCT	9354 - 9374	NS5	-

*Phylogenetic analysis* - Sequences were aligned using Clustal W multiple alignment and edited manually in Bioedit. Phylogenetic relationships were inferred with Bayesian analysis using Markov chain Monte Carlo (MCMC) with BEAST v.1.8.0 under GTR + G + I model. The MCMC chain was run for 10,000,000 generations, sampling every 1,000 generations and a constant coalescent tree prior. The maximum clade credibility tree (MCCT) was chosen from the posterior distribution of 10,001 sampled trees with the program TreeAnnotator version v1.8.0. Statistical support for the inferred Bayesian trees was assessed by posterior probabilities.

*Nucleotide sequences accession numbers* - The nucleotide sequences were deposited on GenBank under the following accession numbers: MF048802-MF048807 for envelope genes, and MF077458-MF07763 NS5 genes.

*Ethical approval* - This study was carried out in accordance with the Declaration of Helsinki as revised in 2000, and approved by the Ethics Committee of the Adolfo Lutz Institute, São Paulo, Brazil. Study participants were not required to provide informed consent as this study was considered by the Ethics Committee to be part of routine surveillance activities.

## RESULTS

Six cell culture samples and one clinical sample (urine) were analysed by means of both ZIKV envelope and NS5 genes amplification, following Sanger sequencing. The presence of inhibitors for ZIKV detection was not evaluated, and no method was used to remove them from urine sample. However, a viral concentration method was employed in this sample. ZIKV envelope and NS5 genes were successfully amplified in cell culture samples, resulting in a specific 1701 pb and 798 pb amplification product, respectively. The ZIKV envelope gene was also effectively amplified from urine samples (concentrated and non-concentrated). However, albeit several attempts to obtain the NS5 fragment from these samples, they were unsuccessful.

**Fig. 1:**
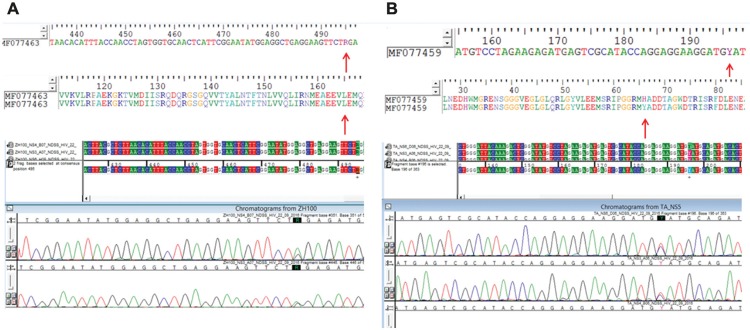
: chromatogram of the partial Zika virus (ZIKV) NS5 region showing ambiguities found in the isolates BR18147/ZH100 (accession number MF077463) and BR31016 (accession number MF077459). (A) Chromatogram and alignment (nucleotide and amino acid) of partial NS5 sequence showing the ambiguity (R = A or G) present in isolate BR18147/ZH100 (MF077463) at position 495. This is a synonymous ambiguity, with both nucleotides coding for Lysine at position 165. (B) Chromatogram and alignment (nucleotide and amino acid) of partial sequence highlighting the ambiguity (Y = C or T) in isolate BR31016 (MF077459) at position 196, which leads to a non-synonymous amino acid substitution at position 66, coding Histidine or Tyrosine.

A total of seven sequences of complete envelope protein (1515 bp) and six sequences of partial NS5 region (667 bp) were obtained in this study. The sequences were aligned using BioEdit sequence alignment editor (version 7.0.5.2) program. ZIKV envelope region showed to be more conserved than the NS5 gene, with a significant lower percentage of nucleotide substitutions (0.26 x 0.95, respectively p = 0.042, Fisher two-tailed). Two NS5 sequences (isolates BR18147/ZH100 and BR31016), presented ambiguities at positions 495 and 196, respectively. The ambiguity found in isolate BR18147/ZH100 (MF077463) is synonymous (R = A or G), with both nucleotides coding for a Lysine at position 165 ( Fig. 1A ); whereas the ambiguity in sample BR31016 (MF077459) (Y = C or T) leads to a non-synonymous amino acid substitution at position 66 (coding for Histidine or Tyrosine) ( Fig. 1B ).

**Fig. 2:**
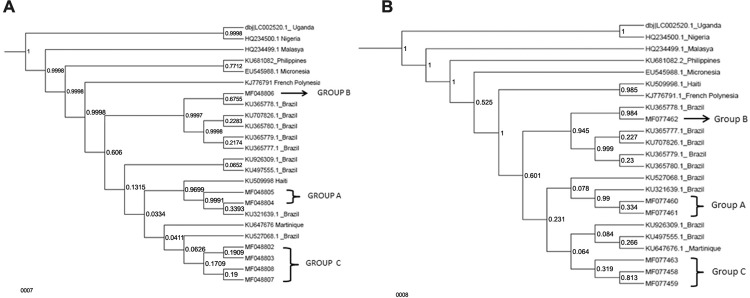
: bayesian phylogenetic tree of Brazilian Zika virus (ZIKV) strains. (A) Bayesian phylogenetic tree of complete envelope nucleotide sequence generate with Markov chain Monte Carlo (MCMC) with BEAST v.1.8.0 under GTR+G+I model of seven Brazilian ZIKV strains (six cell culture and one urine sample). Reference of envelope ZIKV genes was obtained from GenBank database. Capital letters A, B and C represents three different groups. Accession numbers and locality of each strain are indicated. The scale indicates the number of divergent nucleotide residues. The posterior probability of the branch values are indicated at nodes. (B) Bayesian phylogenetic tree of partial NS5 nucleotide sequence generated with Markov chain Monte Carlo (MCMC) with BEAST v.1.8.0 under GTR+G+I model of six Brazilian ZIKV strains (six cell culture samples). Reference of NS5 ZIKV genes was obtained from GenBank database. Capital letters A, B and C represents three different groups. Accession numbers and locality of each strain are indicated. The scale indicates the number of divergent nucleotide residues. The posterior probability of the branch values are indicated at nodes.

Two strains belonging to African lineage (HQ234500 and djLC002500), six strains from Asian lineage (HQ234499, EU545988, KU681082, KU509998, KJ776791 and KU647676), and nine Brazilians strains (KU707826, KU365778, KU365780, KU365779, KU365777, KU926309, KU497555, KU321639, KU527068) were used to infer genetic relationships between the worldwide ZIKV samples and the strains characterised in the present study. The newly identified ZIKV strains in countries of Americas are all close to Asian and Pacific strains as well as the samples characterised in the present study. In addition, both, envelope and NS5 genes of Brazilian ZIKV strains detected here could be discriminated into three clusters phylogenetically distinct, designated A, B and C. Group A is formed by donor and recipient samples (isolates BR22482 and BR17829), Group B is composed by cell culture isolate BR2716, and Group C is constituted by cell culture and urine pair samples (isolate BR31016) and other two distinct cell culture isolates (BR18147/ZH100 and BR19147/23101702) ( Fig. 2A-B ).

According to the identity score provided by BLAST/NCBI, Group A showed 100% of nucleotide identity to two samples previously detected in Recife, Brazil in 2015 (KR872956 and KX197192) ( Donald et al. 2016 ) for both envelope and NS5 genes. Group B exhibited 99% of nucleotide identity (envelope and NS5) to more than 20 ZIKV strains, including samples from French Guiana detected in 2015 (KU758871 and KU758870), and Puerto Rico identified in 2015 (KX087101 and KX601168) and 2016 (KY075934). Group C displayed a homology of 100% at the envelope protein to 17 ZIKV sequences, comprising four strains detected in Nicaragua in 2016 (KY765327, KY765326, KY765325 and KY765324), two strains isolated in Honduras in 2016 (KX262887 and KY785414), six strains from French Polynesia detected in 2013 (KX447519, KX447518, KX447513, KX447510 and KX369547) and 2014 (KX447520), two strains identified in United States in 2016 (KY325479 and KY325465) ( Grubaugh et al. 2017 ), two strains reported in Paraiba, Brazil in 2015 (KX576684 and KX280026), and one strain detected in Rio de Janeiro, Brazil in 2016 (KY014313) ( Metsky et al. 2017 ). Finally, Group C presented a similarity of 99% at the NS5 region to more than 30 strains, highlighting strains isolated in Central America: Peru in 2016 (KY693679), Honduras in 2016 (KY785452), Mexico in 2016 (KY606272), and Ribeirão Preto, Brazil (KY559015).

**Fig. 3:**
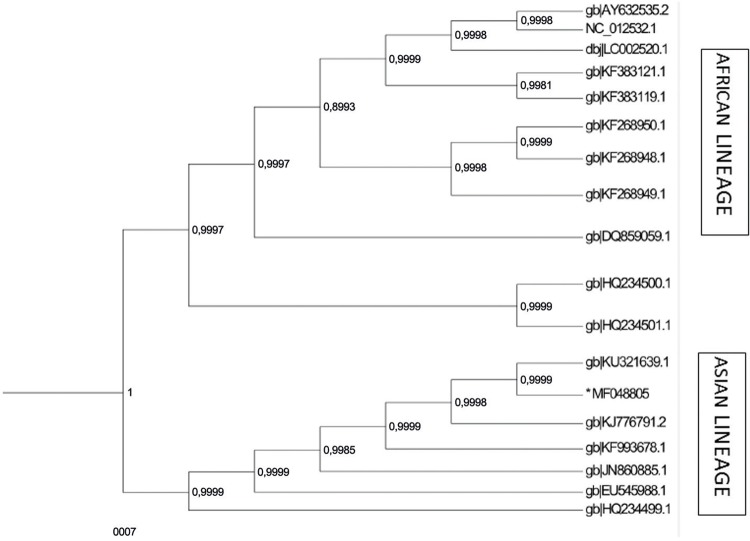
: Sanger population sequence obtained from recipient *MF48805 compared to NGS sequence obtained from the same sample (KU321639). Bayesian phylogenetic tree of complete envelope nucleotide sequence generate with Markov chain Monte Carlo (MCMC) with BEAST v.1.8.0 under GTR+G+I model. Sixteen reference envelope strains were obtained from GenBank database. Lineages assigned for ZIKV strains are indicated on the right. The posterior probability of the branch values are indicated at nodes. Clades with high posterior probability (i.e. ≥ 0.95) were considered highly supported.

To evaluate the informative potential of Sanger population sequences compared to NGS sequences, and to verify if the region here sequenced are useful to provide phylogenetic information suitable to molecular epidemiology studies, we used the recipient sequence generated by Barjas-Castro et al. (2016) using NGS (KU321639) and the recipient sequence generated here using Sanger sequencing (MF48805). A 1515 nucleotide analysis revealed that the two samples were 100% identical and as expected clustered together in a monophyletic branch with high posterior probability (i.e. ≥ 0.95). The partial ZIKV sequences generated by Sanger in this study were evaluated among well described African and Asian lineages reference sequences, showing good support to discriminate this lineages ( Fig. 3 ).

## DISCUSSION

One of the potential threats to public health microbiology in 21st century is the morbidity caused by ZIKV. The severity of ZIKV infection urged World Health Organization (WHO) to declare this virus as a global concern ( Shankar et al. 2017 ). The rapid geographic expansion of ZIKV, genetic diversity, multiple transmission pathways, adaptability to infect distinct vectors, and its association with severe neurological diseases has highlighted a need for robust molecular tools that can be used to efficiently and quickly detect and characterise ZIKV genomes ( Leguia et al. 2017 ). Here we described a targeted RT-PCR amplification and Sanger sequencing strategy developed for complete envelope ZIKV and partial NS5 genes, considered potential drug and vaccine targets ( Shankar et al. 2017 ). The main purpose of that strategy was to introduce easy laboratory procedures in order to handle ZIKV samples and reliably generate genome sequence data in a quick and cost effective manner.

Our primers were designed based on an alignment of 15 ZIKV reference sequences. The references were selected in order to include the diversity of ZIKV strains, and to avoid biasing ZIKV strains that had undergone multiple passages in viral culture or isolated from non-human hosts were not included. The primers have been validated with a scarce set of cell culture and clinical (urine) samples collected during 2016 from a restrict area in Brazil. Although samples for other lineages were not tested, primer sets were designed using global references, so it is likely that it can be used to successfully amplify a myriad of samples.

Conventional one-step RT-PCR has been successfully used for amplification of ZIKV genes ( Faye et al. 2008 ). We developed here a nested RT-PCR. The option for this approach was based on: (i) sensitivity can be further increased, (ii) avoid further amplification of primer-dimer artifacts or nonspecific products generated in the first round, and (iii) a different set of primers could be employed in the second round ( Goode et al. 2002 ). This protocol showed to be effective for ZIKV amplification from cell culture supernatants, but only partially for clinical samples. Nested RT-PCR method did not allow the NS5 amplification in urine sample (both concentrated and non-concentrated). The presence of possible PCR inhibitors in urine was not evaluated in the present study, and they might be hindered the NS5 amplification. Internal controls are necessary for future optimisation of RNA extraction and amplification procedures. A non-amplified NS5 protein could also indicate degradation of the nucleic acid in the urine sample or an unusual sequence. Mismatches in the primer-binding region are known to affect amplification. However the NS5 genomic sequence is highly conserved among Asian lineage strains worldwide ( Metsky et al. 2017 ), suggesting that mismatches were not the probable cause of non-amplification. It is worth to mentioning that only one clinical sample was tested here, and it is important to further evaluate the performance of the present protocol using additional clinical samples, including serum, saliva and urine.

The majority of ZIKV sequences available were obtained using NGS ( Barjas-Castro et al. 2016 , Leguia et al. 2017 , Metsky et al. 2017 ), and this methodology provide valuable information on viral diversity, being pivotal in the analysis of viral quasispecies ( van Boheemen et al. 2017 ). However this tool may be cost effective in specialised core laboratories working with high quality samples and bioinformatics support, a situation not commonly available in clinical and public health laboratories, especially in resource constrained settings. The use of cell culture isolates obtained from small serum samples and the nested RT-PCR followed by Sanger sequencing presented here was a suitable low-cost methodology to sequenced relevant regions of ZIKV genome. Moreover, in the context of outbreaks, where high numbers of samples need to be processed quickly and accurately, these types of tailored strategies can significantly impact operations ( Leguia et al. 2017 ).

It is well known that Sanger sequencing may not have the sensibility to detect minor variants of the RNA viruses quasispecies; nevertheless is an alternative tool, easy to use, robust, affordable, rapid and specific to obtain sequences from the major variant. Thus, it may be an important alternative methodology to NGS. In the present study we were able to demonstrate that major variant of ZIKV envelope gene identified in the recipient transfusion patient ( Barjas-Castro et al. 2016 ) could be recognised by both NGS and Sanger methodologies.

Similarly to the sequences described in the recent widespread epidemic of ZIKV in the Americas, the partial genome sequences characterised in this study clustered with the Asian clade, covering sequences from New World, Pacific, Micronesian and Malaysian strain ( Faria et al. 2016 , Metsky et al. 2017 ). ZIKV envelope protein is responsible for virus entry and represents a major target for neutralising antibodies. On the other hand, NS5 is critical for ZIKV replication. Therefore, envelope glycoprotein and NS5 polymerase are major targets for ZIKV antiviral and vaccine developments ( Shankar et al. 2017 ). Nucleotide ambiguities were identified in NS5 region in two sequences analysed here (isolates BR18147/ZH100 and BR31016). The precise impact of amino acid changes cannot be predicted from sequence information alone and studies attempting to correlate nucleotide differences with antigenic differences are extremely important ( Dai et al. 2016 )

In conclusion, the present study provided a simple and low-cost Sanger protocol to sequence relevant genes of the ZIKV genome able to provide robust phylogenetic signals that allow molecular epidemiological studies.

*Sequence data* - Sequences are available at GenBank with accession numbers: MF048802 to MF048807 and MF077458 to MF07763.
